# Psychosocial work environment and emotional exhaustion among middle-aged employees

**DOI:** 10.1186/1756-0500-4-101

**Published:** 2011-04-04

**Authors:** Minna Helkavaara, Peppiina Saastamoinen, Eero Lahelma

**Affiliations:** 1 Department of Public Health, University of Helsinki, Helsinki, Finland

## Abstract

**Background:**

This study examined the associations of job control, organizational justice and bullying at the workplace with emotional exhaustion. This was done by adjusting firstly for age and occupational class, secondly physical work factors, thirdly mutually adjusting for the three psychosocial factors and fourthly adjusting for all studied variables simultaneously.

Data were derived from the Helsinki Health Study baseline surveys conducted in 2001 and 2002, including 40-60-year-old employees of the City of Helsinki (n = 5819, response rate 66%). Exhaustion was measured with a six-item subscale from Maslach Burnout Inventory (MBI). Psychosocial factors included Karasek's job control, organizational justice and bullying at the workplace. Logistic regression analysis was used.

**Results:**

Among women 23% and among men 20% reported symptoms of emotional exhaustion. Among women all psychosocial factors were associated with exhaustion when adjusted for age and occupational class as confounders. When physical work factors were additionally adjusted for, the associations slightly attenuated but remained. When psychosocial work factors were simultaneously adjusted for each other, their associations with exhaustion attenuated but remained. Among men all psychosocial factors were associated with exhaustion when adjusted for confounders only. When adjusted for physical work factors the associations slightly attenuated. When psychosocial factors were simultaneously adjusted for each other, associations of organizational justice and bullying with exhaustion attenuated but remained whereas job control lost its association.

**Conclusions:**

Identifying risk factors for emotional exhaustion is vital for preventing subsequent processes leading to burnout. Psychosocial factors are likely to contribute to exhaustion among female as well as male employees. Thus management and occupational health care should devote more attention to the psychosocial work environment in order to be able to prevent exhaustion and burnout at the workplaces.

## Background

Burnout is a widespread health-related problem in current working life and for example in Finland it is estimated that 25 percent of employees are suffering from mild symptoms of burnout and 2,5 percent of employees are suffering from burnout [1]. Burnout develops as a prolonged response to chronic emotional and interpersonal stressors as these appear repeatedly in the employees' work environment [2]. Burnout contains three dimensions including emotional exhaustion, cynicism and lack of professional efficacy [3]. It is widely agreed that the key dimension of burnout is exhaustion [2,4-7]. Exhaustion may persist even for several years [8], and have synchronous effects with cynicism [9]. Professional inefficacy has more complicated associations with the two other dimensions of burnout. In some studies reduced personal accomplishment has had direct associations with exhaustion and cynicism, but in some others it has been a more independent dimension [10,11].

This study focuses on exhaustion among middle-aged employees. Previous studies suggest that a number of work characteristics such as high job demands, long working hours, high workload and time pressures are likely to contribute to exhaustion [2,7,12]. Exhaustion also has health-related consequences. For example a Dutch study found that among various psychological problems burnout was the most common reason for sickness absence and work disability [12].

As exhaustion is at the core of burnout, it is important to add our understanding of the psychosocial and other factors in the work environment that may contribute to exhaustion among employees. The job demand-control model by Karasek (1979) [13] is often used in studies examining work-related psychosocial exposures. Job demands act as psychological stressors in the work environment whereas job control focuses on employees' ability to control their own activities and skill usages. While high job demands have been found to be associated with high levels of exhaustion [7,11], associations of job control with exhaustion have been weaker [14]. Structural models of burnout have shown that job control is more strongly associated with high levels of disengagement but less with exhaustion [11]. However, strong relationships between role conflicts and exhaustion have been confirmed [15].

Recently, further work-related psychosocial factors have been included in health research, devoting attention to management styles and the workplace climate. Organizational justice focuses on the fairness of decision making in organizations and the treatment of individuals by their supervisors. Presence of bullying reflects the social atmosphere at workplaces. Organizational injustice and bullying at the workplace have been found to be associated with various mental and physical health problems [16-19]. However, we lack studies using a broad psychosocial framework including both several less used and established factors with regard to exhaustion among employees.

The relative significance of heavy physical demands has tended to decline in the work life, but many jobs still contain physical demands and these continue to be associated with various health-related problems [20]. It has been emphasised that the essence of exhaustion is more mental than physical fatigue [2]. Nevertheless also physical work factors play a role and they too need to be considered when examining the association between psychosocial factors and exhaustion.

Our first aim was to examine the associations of each psychosocial factor, i.e. job control, organizational justice and bullying at the workplace with exhaustion, adjusting for age and occupational class as confounders. Our second aim was to examine whether physical work factors affect the associations of psychosocial factors with exhaustion. Our third aim was to examine the associations while mutually adjusting the three psychosocial factors to be able to find out their own and shared associations with exhaustion. We finally examined the associations of the psychosocial factors with exhaustion simultaneously adjusting for all studied variables to be able to find out the independent effects of the psychosocial factors.

## Methods

### Sample and procedure

This study is part of the Helsinki Health Study examining health and well-being among ageing employees of the City of Helsinki, Finland. With nearly 40.000 employees the City of Helsinki is the largest employer in the country. Employment sectors include education, social and health care, cultural services, public transportation, general administration, environmental and technical services with a wide range of different occupations and work tasks.

This study was based on two cross-sectional baseline surveys conducted in 2001 and 2002. A self-administered questionnaire was mailed to each employee, who during the year of each survey reached the age of 40, 45, 50, 55 or 60. Participation to this survey was voluntary and the participants were informed of this. The data include 4.674 women and 1.145 men. The data are held at the University of Helsinki and are not openly available. The response rate to this study was 66%.

### Measures

#### Exhaustion

Emotional exhaustion was measured by using an inventory which has been developed as a subscale from the Maslach Burnout Inventory (MBI) [3] at the Finnish Institute of Occupational Health [8]. Questions concerning work with clients and one question concerning job frustration were omitted from MBI. Consequently the inventory included the following six items measuring exhaustion: 1) I feel totally worn out after a day at work, 2) I feel tired in the morning when I have to get up and go to work, 3) I have to work too hard, 4) I feel like I'm totally exhausted, 5) My work is definitely too stressful, and 6) I worry about my work even when I am off duty. Response alternatives ranged on a five-point scale from very seldom to very often. First the response scales were dichotomized. Those who reported exhaustion very seldom, seldom or sometimes received the value of 0. Those who reported exhaustion often or very often received the value of 1. Second a sum score was calculated over all six items. The final exhaustion sum score ranged from 0 to 6. In the present study the cut-off point was set between scores 2 and 3. Scores 0-2 indicate no or low exhaustion and scores 3-6 indicate high exhaustion. Cronbach's alpha for the exhaustion scale was .89.

#### Job control

The job control dimension from Karasek's (1979) [13] demand-control model was used. Job control was measured by nine items (Cronbach's alpha .82). The scale was weighted according Karasek's procedure [21] and divided into quartiles. The lowest quartile signifies low control and is considered most harmful while the highest quartile signifies beneficial high job control. The job demands dimension of Karasek's model measures work related stress and our measure of exhaustion also includes three items measuring work related stress. The correlation between job demands and exhaustion was relatively high, i.e. 52 for women and .49 for men. Since there is potential conceptual overlap between job demands and exhaustion we did not include the job demands dimension to our analyses.

#### Organizational justice

Organizational justice was measured by two subscales: procedural and relational justice [22,23]. Procedural justice is associated with the fairness of decision making process in the organization. Relational justice is associated with supervisor's general behaviour. Both scales included four items on a five-point scale. Response alternatives varied from strongly agree to strongly disagree. We performed principal component analysis (PCA) to reconfirm the construct validity of the measure and received a one component solution, instead of a two component solution. The correlation between the two scales was r =.70. Based on these analyses the two scales were combined into one summary measure of organizational justice which was divided into quartiles (Cronbach's alpha .91). Our focus was on low justice, i.e. organizational injustice.

#### Bullying at the workplace

Witnessing the presence of bullying at one's workplace was asked with a question introduced as follows: "Psychological harassment or workplace bullying means social isolation of a member of a work community, threatening, talking behind one's back or other kind of pressurizing" [16,24]. The respondents were then asked whether they had witnessed such behaviour in their work unit or department. The response alternatives were: never, sometimes, repeatedly, and cannot say. Similar measurement has been used in previous studies as well [25,26].

#### Physical work factors

**Figure 1:**
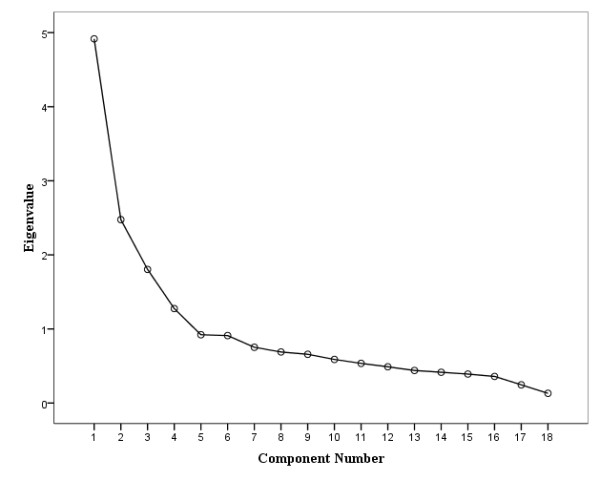
**Scree plot of eigenvalues from principal component analysis of physical work factors**. A scree-plot reveals the optimal number of components in PCA.

The questionnaire contained 18 questions measuring the physical work factors [27]. The response alternatives were: does not exist, exists but does not cause harm, and exists and causes harm. Principal component analysis (PCA) was used to compress the information. Component loadings are presented in Table 1 and a scree-plot of associated eigenvalues in Figure 1. These show that PCA produced four components: 1) 'work including physical and chemical exposures' such as noise, solvents, heat, dust and humidity (Cronbach's alpha .75); 2) 'physically strenuous work' including factors such as awkward postures, rotation of back, repetitive movements, and lifting or carrying heavy loads (Cronbach's alpha .79); 3) 'office work' including sitting and working with computer (Cronbach's alpha .84); and 4) 'work in upright position' including standing and walking (Cronbach's alpha .81). Component sum variables were calculated from component items.

**Table 1:** Principal component analysis of physical work factors

Items	1 Work including physical and chemical exposures	2 Physically strenuous work	3 Office work	4 Working in upright position
Awkward postures		0.841		
Rotation of back		0.853		
Repetitive movements		0.805		
Lifting or carrying heavy loads		0.575		
Sitting			0.668	
Working with display terminal			0.908	
Working with computer mouse			0.895	
Standing				0.878
Walking				0.804
Noise	0.422			
Vibration	0.590			
Weak or distracting lighting	0.642			
Gases or irritant substances	0.523			
Heat, chilliness, draft or changes of temperature	0.661			
Dryness of air	0.574			
Dust and dirtiness	0.739			
Wetness and humidity	0.682			
Mould	0.546			

Component loadings.

#### Confounders

The analyses were adjusted for age (40, 45, 50, 55 and 60 years) and occupational class which included six hierarchical classes: unskilled manuals, skilled manuals, routine non-manuals, semi-professionals, professionals and managers [28].

### Statistical methods

Firstly prevalence data for exhaustion by independent variables were calculated and chi square tests used. Secondly logistic regression analysis was used to examine the association of psychosocial factors and other independent variables with exhaustion. Four models were fitted with exhaustion as the dependent variable: 1) In the base model each psychosocial factor was adjusted for age and occupational class only, 2) physical work factors were then added to the base model, 3) all three psychosocial variables were next mutually adjusted for adding them simultaneously to the base model, and 4) finally all studied variables were included into the fully adjusted model. The results from the models are presented as odds ratios (OR) and their 95% confidence intervals (CI). All analyses were conducted separately for women and men as there are differences in the work tasks, organizational positions and employment sectors between the two genders. SPSS version 15.0 was used.

### Ethical approval

The Helsinki Health Study protocol has been approved by ethics committees of the Department of Public Health, University of Helsinki, and the City of Helsinki health authorities, Finland.

## Results

Among women 23% reported exhaustion (Table 2). Women who were 55 years old and those in the highest occupational classes more often than others reported exhaustion. Women reporting low job control, organizational injustice and repeatedly witnessing bullying at the workplace showed a high prevalence of exhaustion. Among men 20% reported exhaustion. Men in higher occupational classes reported more exhaustion than other men. As for women also for men those reporting organizational injustice and bullying showed a high prevalence of exhaustion.

**Table 2:** The prevalence of emotional exhaustion by age, occupational class and psychosocial work factors (%)

	Symptoms of emotional exhaustion			
	
	Women		Men	
	No. of cases	%	No. of cases	%
Age (years)				
40	172	19.3	37	19.2
45	187	19.4	45	21.0
50	213	22.4	47	20.5
55	296	26.8	55	19.4
60	127	25.0	35	21.2
		***		Ns.
Occupational class				
Unskilled manuals	89	22.4	28	15.8
Skilled manuals	30	23.3	12	12.4
Routine non-manuals	349	18.8	21	19.1
Semi-professionals	205	24.3	44	19.2
Professionals	242	26.7	59	21.2
Managers	80	28.6	55	28.5
		***		Ns.
Job control				
High	252	20.6	62	20.7
Rather high	205	18.9	57	18.1
Rather low	214	19.8	38	17.9
Low	324	31.6	62	24.0
		***		Ns.
Organizational justice				
High	145	13.8	27	12.5
Rather high	228	17.6	56	15.8
Rather low	267	23.4	53	19.2
Low	355	38.4	83	35.0
		***		***
Bullying at workplace				
Not at all	243	15.7	66	14.4
Sometimes	538	23.8	100	20.3
Repeatedly	159	43.7	34	42.5
Don't know	55	22.8	19	34.5
		***		***
Total N	4674		1145	

** p < 0.01, *** p < 0.001 (χ^2^test).

The associations of psychosocial factors with exhaustion were next examined using logistic regression analysis (Table 3). After adjusting for age and occupational class as confounders, low job control was clearly associated with exhaustion (OR 2.65, 2.13-3.30) among women. Also organizational injustice (OR 4.02, 3.22-5.02) and repeatedly witnessing bullying at one's workplace (OR 4.40, 3.42-5.66) were associated with exhaustion. Adjusting for physical work factors only slightly weakened the associations. When all three psychosocial factors were simultaneously adjusted for each other their associations with exhaustion were weakened but nevertheless remained. Adjusting for all variables in the full model did not further affect the associations found in the previous model.

**Table 3:** Associations of psychosocial factors with emotional exhaustion among women

Women	Adjusted for age and occupational class OR (95% CI)	Adjusted for age, occupational class and physical work factors OR (95% CI)	Adjusted for age, occupational class and psychosocial work environment OR (95% CI)	Fully adjusted model OR (95% CI)
Job control				
High	1.00	1.00	1.00	1.00
Rather high	1.00 (0.81-1.23)	0.98 (0.79-1.21)	0.92 (0.74-1.14)	0.92 (0.74-1.14)
Rather low	1.22 (0.98-1.51)	1.14 (0.92-1.42)	1.01 (0.80-1.26)	0.98 (0.79-1.23)
Low	2.65 (2.13-3.30)	2.47 (1.97-3.08)	1.79 (1.42-2.26)	1.73 (1.36-2.18)
Organizational justice				
High	1.00	1.00	1.00	1.00
Rather high	1.32 (1.05-1.66)	1.28 (1.02-1.61)	1.21 (0.96-1.53)	1.17 (0.93-1.48)
Rather low	1.96 (1.57-2.45)	1.86 (1.49-2.34)	1.61 (1.27-2.04)	1.55 (1.22-1.96)
Low	4.02 (3.22-5.02)	3.70 (2.95-4.63)	2.61 (2.03-3.35)	2.53 (1.98-3.25)
Bullying at workplace				
Not at all	1.00	1.00	1.00	1.00
Sometimes	1.74 (1.47-2.06)	1.61 (1.35-1.91)	1.39 (1.16-1.66)	1.30 (1.09-1.56)
Repeatedly	4.40 (3.42-5.66)	4.00 (3.10-5.16)	2.37 (1.79-3.13)	2.25 (1.70-2.97)
Don't know	1.57 (1.12-2.19)	1.52 (1.09-2.13)	1.29 (0.92-1.82)	1.31 (0.93-1.84)

Odds ratios (OR) from logistic regression analysis.

The results for men were mostly confirmed those found for women. In the base model, adjusting for age and occupational class, low job control (OR 2.11, 1.31-3.37), organizational injustice (OR 4.72, 2.86-7.80) and repeatedly witnessing bullying at the workplace (OR 5.51, 3.23-9.40) were all clearly associated with exhaustion (Table 4). Adjusting for physical work factors slightly weakened the associations of each psychosocial factor with exhaustion. When all three psychosocial work factors were mutually adjusted for their associations attenuated somewhat but remained except for job control which lost statistical significance. In the fully adjusted model the associations for organizational justice and bullying were further slightly attenuated but remained.

**Table 4:** Associations of psychosocial factors with emotional exhaustion among men

Men	Adjusted for age and occupational class OR (95% CI)	Adjusted for age, occupational class and physical work factors OR (95% CI)	Adjusted for age, occupational class and psychosocial work environment OR (95% CI)	Fully adjusted model OR (95% CI)
Job control				
High	1.00	1.00	1.00	1.00
Rather high	0.93 (0.62-1.39)	0.90 (0.60-1.36)	0.81 (0.53-1.23)	0.84 (0.55-1.28)
Rather low	1.13 (0.70-1.80)	1.08 (0.67-1.74)	0.87 (0.53-1.42)	0.86 (0.52-1.41)
Low	2.11 (1.31-3.37)	2.05 (1.27-3.31)	1.26 (0.76-2.09)	1.32 (0.79-2.21)
Organizational justice				
High	1.00	1.00	1.00	1.00
Rather high	1.45 (0.88-2.39)	1.38 (0.83-2.29)	1.41 (0.85-2.37)	1.32 (0.79-2.20)
Rather low	1.98 (1.18-3.31)	1.80 (1.07-3.03)	1.78 (1.04-3.05)	1.59 (0.93-2.73)
Low	4.72 (2.86-7.80)	4.10 (2.46-6.84)	3.34 (1.92-5.78)	2.85 (1.64-4.96)
Bullying at workplace				
Not at all	1.00	1.00	1.00	1.00
Sometimes	1.59 (1.13-2.25)	1.43 (1.01-2.03)	1.27 (0.88-1.83)	1.16 (0.80-1.66)
Repeatedly	5.51 (3.23-9.40)	4.80 (2.79-8.27)	2.91 (1.60-5.26)	2.61 (1.43-4.75)
Don't know	3.42 (1.83-6.39)	3.35 (1.78-6.30)	2.69 (1.41-5.13)	2.56 (1.33-4.91)

Odds ratios (OR) from logistic regression analysis.

Additionally interactions were tested but not found between age and occupational class (p < .07 for women, p < .71 for men).

## Discussion

This study sought to examine the own and mutually adjusted associations of psychosocial work factors and physical work factors with emotional exhaustion among middle-aged municipal employees of the City of Helsinki. This was done by adjusting first for age and occupational class as confounders, second for physical work factors, third simultaneously for all psychosocial factors and finally simultaneously for all work related factors.

Our results showed that each psychosocial work factor, i.e. job control, organizational injustice and presence of bullying at workplace, was associated with exhaustion. Adjusting for physical work factors had only minor effects on these associations. Among women the associations remained robust even after adjustments for confounders, physical work factors as well as mutual adjustment for the three psychosocial work factors. The results were otherwise similar for both women and men but for men job control lost its association after mutual adjustment for the three psychosocial work factors. Full adjustment for all work related factors did not further affect the associations of mutually adjusted psychosocial work factors with emotional exhaustion.

Karasek's [13] job control is often included in occupational studies. In our study it was associated with exhaustion throughout the analyses only among women. In previous studies job control has shown associations with other mental and physical health problems [20,29]. Among men associations of job control with exhaustion could be found after adjusting for physical work factors but no more after mutual adjustments for all psychosocial factors. The much smaller number of men compared to women in this study needs to be considered. However, our results suggest that among men job control is interrelated with organizational injustice and bullying at workplace. Job control may thus have a dissimilar significance to exhaustion in men compared to some other mental and physical health problems [20,29].

Organizational injustice was strongly associated with exhaustion among both women and men. This is in line with earlier findings where unfair treatment in organization has been a risk for employees' health. Organizational injustice has been associated with poor health, psychological distress, smoking and sickness absence [17,23,30,31]. A longitudinal study found that an adverse change in relational justice was followed by an increased risk of poor health [17]. In contrast a favorable change in relational justice was followed by a lowered risk of poor health. Another longitudinal study found that the domain of fairness in organization is a critical factor when predicting upcoming burnout among employees [10]. If there were problems with fairness in the workplace, employees with symptoms of exhaustion were likely to develop burnout over time. In our study overall organizational injustice was likely to be a risk factor for emotional exhaustion, but this finding needs to be further confirmed.

Repeatedly witnessing bullying at the workplace was also strongly associated with exhaustion among both women and men. In previous studies bullying at the workplace has been associated with mental stress reactions as well as symptoms of anxiety and depression [18,25,32]. Bullying has also been found to be associated with somatic complaints and pain [19,33]. Bullying at the workplace has further been associated with lack of support from superior and an authoritarian way of settling conflicts [25,34]. Our results suggest that an adverse organizational climate allowing bullying to take place provides a risk for emotional exhaustion.

Physical work factors only minimally weakened the associations of psychosocial work factors with exhaustion. This confirms previous results showing that the foundations of exhaustion are likely to be in the mental and psychosocial domain rather than the physical domain of work exposures [2].

Our study contributes to a broader psychosocial view about the factors associated with emotional exhaustion among employees. Low job control was a consistent risk factor for exhaustion among women only and its strength among the three studied psychosocial factors was the weakest for both women and men. The further psychosocial stressors, i.e. organizational injustice and bullying at the workplace, showed particularly strong associations with exhaustion among both women and men.

### Limitations and strengths

The data were collected in early 2000s and after that changes may have taken place at the workplaces and among the studied employees. Our study was cross-sectional and explorative in nature. Causal relationships between psychosocial work factors and exhaustion cannot be established. The associations could be both ways, although the existing longitudinal evidence would support psychosocial factors preceding and contributing to adverse health-related outcomes, including burnout.

The response rate to our survey was 66% which is typical of this kind of questionnaire surveys. According to our non-response analyses using register based data men, younger people and those in lower socioeconomic positions were somewhat underrepresented among the respondents [35]. However, the differences between the respondents and non-respondents were minor and in the non-response analyses these differences did not cause bias when the association between sickness absence and other factors was examined. Thus, it is unlikely that the differences in the non-response could substantially bias the associations examined in this study. Nevertheless, we acknowledge that non-response was substantial and that needs to be kept in mind when drawing conclusions from the analyses.

All our information was based on self-reports from questionnaires and reporting bias and dispositions to respond as well as personality traits may have affected the results. People with negative affectivity might assess their working conditions in more adverse terms [36]. This kind of responding dispositions might overestimate the observed associations. In contrast, associations may be underestimated if people with health problems have left work life prior our survey.

Since our data represent middle-aged municipal employees, generalization of the current findings to private sector employees or to younger employees cannot be made.

The strengths of this study include a large data source from 2001-2002 with both women and men from a large variety of occupations. Additionally our data contained several psychosocial factors as well as physical work factors.

## Conclusions

Our results confirm that psychosocial factors in the work environment are associated with emotional exhaustion among women and men. Factors, such as organizational justice and bullying turned out to be particularly strongly associated with exhaustion. Thus managerial procedures and treatment of employees in organization should be taken into account at workplaces while planning and implementing measures aiming at promoting employee health and well-being.

## Competing interests

The authors declare that they have no competing interests.

## Authors' contributions

MH, EL and PS planned the study. MH conducted the statistical analyses and drafted the text. EL and PS commented and contributed to the final draft. All authors read and approved the final manuscript.

## References

[B1] Ahola K Honkonen T Isometsä E Kalimo R Nykyri E Aromaa A Lönnqvist J The relationship between job-related burnout and depressive disorders - results from the Finnish Health 2000 Study J Affect Disord 2005 88 55 62 10.1016/j.jad.2005.06.004 16038984

[B2] Maslach C Schaufeli WB Leiter MP Job burnout Annu Rev Psychol 2001 52 397 422 10.1146/annurev.psych.52.1.397 11148311

[B3] Maslach C Jackson SE The measurement of experienced burnout J Occup Behav 1981 2 99 113 10.1002/job.4030020205

[B4] Shirom A Cooper CL, Robertson IT Burnout in work organizations International review of industrial and organizational psychology 1989 New York: Wiley 25 48

[B5] Koeske GF Koeske RD Construct validity of the Maslach Burnout Inventory: A critical review and reconceptualization J App Behav Sci 1989 25 131 144 10.1177/0021886389252004

[B6] Buunk BP Schaufeli WB Schaufeli WB, Maslach C, Marek T Burnout: A perspective from social comparison theory Professional burnout: Recent developments in theory and research 1993 Washington DC: Taylor & Francis 53 69

[B7] Bakker AB Schaufeli WB Sixma HJ Bosveld W Van Dierendonck D Patient demands, lack of reciprocity, and burnout: A five-year longitudinal study among general practitioners J Organiz Behav 2000 21 425 441 10.1002/(SICI)1099-1379(200006)21:4<425::AID-JOB21>3.0.CO;2-#

[B8] Toppinen-Tanner S Kalimo R Mutanen P The process of burnout in white-collar and blue-collar jobs: eight-year prospective study of exhaustion J Organiz Behav 2002 23 555 570 10.1002/job.155

[B9] Maslach C Leiter MP Cooper CL Stress and burnout: The critical research Handbook of stress medicine and health 2005 London: CRC Press 153 170

[B10] Maslach C Leiter MP Early predictors of job burnout and engagement J Appl Pschol 2008 93 498 512 10.1037/0021-9010.93.3.498 18457483

[B11] Demerouti E Bakker AB Nachreiner F Schaufeli WB The Job demands-resources model of burnout J Appl Psychol 2001 86 499 512 10.1037/0021-9010.86.3.499 11419809

[B12] Bekker MHJ Croon MA Bressers B Childcare involvement, job characteristics, gender and work attitudes as predictors of emotional exhaustion and sickness absence Work & Stress 2005 19 221 237

[B13] Karasek RA Job demands, job decision latitude, and mental strain: implications for job redesign Admin Sci Q 1979 24 285 308 10.2307/2392498

[B14] De Jonge J Bosma H Peter R Siegrist J Job strain, effort-reward imbalance and employee well-being: a large-scale cross-sectional study Soc Sci & Med 2000 50 1317 1327 10.1016/s0277-9536(99)00388-3 10728851

[B15] Cordes CL Dougherty TW A review and an integration of research on job burnout Acad Management Rev 1993 18 621 656 10.2307/258593

[B16] Kivimäki M Elovainio M Vahtera J Workplace bullying and sickness absence in hospital staff Occup Environ Med 2000 57 656 660 10984336 10.1136/oem.57.10.656 PMC1739873

[B17] Kivimäki M Ferrie JE Head J Shipley MJ Vahtera J Marmot MG Organisational justice and change in justice as predictors of employee health: the Whitehall II Study J Epidemiol Community Health 2004 58 931 937 15483310 10.1136/jech.2003.019026 PMC1732612

[B18] Vartia M Consequences of workplace bullying with respect to the well-being of its targets and the observers of bullying Scand J Work Environ Health 2001 27 63 69 11266149 10.5271/sjweh.588

[B19] Saastamoinen P Laaksonen M Leino-Arjas P Lahelma E Psychosocial risk factors of pain among employees Eur J Pain 2009 13 102 108 10.1016/j.ejpain.2008.03.006 18440254

[B20] Laaksonen M Rahkonen O Martikainen P Lahelma E Associations of psychosocial working conditions with self-rated general health and mental health among municipal employees Int Arch Occup Environ Health 2006 79 205 212 10.1007/s00420-005-0054-7 16254726

[B21] Karasek RA Job content questionnaire and user's guide. Revision 1.1 1985 Department of Work Environment, University of Massachusetts, Lowell, MA

[B22] Moorman RH Relationship between organizational justice and organizational citizenship behaviors: Do fairness perceptions influence employee citizenship? J Appl Psychol 1991 76 845 855 10.1037/0021-9010.76.6.845

[B23] Kivimäki M Elovainio M Vahtera J Ferrie JE Organizational justice and health of employees: prospective cohort study Occup Environ Med 2003 60 27 34 12499453 10.1136/oem.60.1.27 PMC1740369

[B24] Lehto AM Sutela H Uhkia ja mahdollisuuksia. Työolotutkimusten tuloksia 1977-2003. Tilastokeskus 2004 Helsinki: Edita Prima

[B25] Hansen ÅM Hogh A Persson R Karlson B Garde AH Ørbæk P Bullying at work, health outcomes, and physiological stress response J Psychosom Res 2006 60 63 72 10.1016/j.jpsychores.2005.06.078 16380312

[B26] Kivimäki M Virtanen M Vartia M Elovainio M Vahtera J Keltikangas-Järvinen L Workplace bullying and the risk of cardiovascular disease and depression Occup Environ Med 2003 60 779 783 14504368 10.1136/oem.60.10.779 PMC1740404

[B27] Piirainen H Hirvonen M Elo AL Huuhtanen P Kandolin I Kauppinen K Ketola R Lindström K Salminen S Reijula K Toivanen M Viluksela M Virtanen S Work and health - interview study 2000 [in Finnish] 2000 Helsinki: Finnish Institute of Occupational Health

[B28] Lahelma E Martikainen P Rahkonen O Roos E Saastamoinen P Occupational class inequalities across key domains of health: Results from the Helsinki Health Study Eur J Pub Health 2005 15 504 510 10.1093/eurpub/cki022 16014660

[B29] Bosma H Peter R Siegrist J Marmot M Two alternative job stress models and the risk of coronary heart disease Am J Pub Health 1998 88 68 74 10.2105/AJPH.88.1.68 PMC1508386 9584036

[B30] Kouvonen A Vahtera J Elovainio M Cox SJ Cox T Linna A Virtanen M Kivimäki M Organisational justice and smoking: the Finnish public sector study J Epidemiol Community Health 2007 61 427 433 10.1136/jech.2007.061739 17435210 PMC2465689

[B31] Head J Kivimäki M Siegrist J Ferrie JE Vahtera J Shipley MJ Marmot MG Effort-reward imbalance and relational injustice at work predict sickness absence: The Whitehall II study J Psychosom Res 2007 63 433 440 10.1016/j.jpsychores.2007.06.021 17905053

[B32] Niedhammer I David S Degioanni S Association between workplace bullying and depressive symptoms in the French working population J Psychosom Res 2006 61 251 259 10.1016/j.jpsychores.2006.03.051 16880029

[B33] Einarsen S Raknes BI Matthiesen SB Hellesøy OH Helsemessige aspekter ved mobbing I arbetslivet Nordisk Psykologi 1996 48 116 137

[B34] Vartia M The sources of bullying - psychosocial work environment and organizational climate Eur J Work Org Psychol 1996 5 203 214 10.1080/13594329608414855

[B35] Laaksonen M Aittomäki A Lallukka T Rahkonen O Saastamoinen P Silventoinen K Lahelma E Register-based study among employees showed small nonparticipation bias in health surveys and check-ups J Clin Epidemiol 2008 61 900 906 10.1016/j.jclinepi.2007.09.010 18486445

[B36] Watson D Pennebaker JW Health complaints, stress, and distress: exploring the central role of negative affectivity Psychol Rev 1989 96 234 54 10.1037/0033-295X.96.2.234 2710874

